# Supplementary material for: Sensing and seeing associated with overlapping occipitoparietal activation in simultaneous EEG-fMRI
Source: Neurosci Conscious. 2021 Jun 21;2021(1):niab008. doi: 10.1093/nc/niab008 (PMC8216203; doi:10.1093/nc/niab008)
Supplement: niab008_Supplementary_Data [file niab008_supplementary_data.pdf]

Neuroscience of CONSCIOUSNESS

OPEN SCIENCE BADGE APPLICATION FORM

Open Data Badge

Please provide the URL, DOI, or other permanent path for accessing the data in a public, open access repository.

Scrivener, C. L., Malik, A., Lindner, M., & Roesch, E. B. (2020, May 24). Sensing and seeing associated with overlapping occipitoparietal activation in simultaneous EEG-fMRI. https://doi.org/10.17605/OSF.IO/W6BH3

Is there sufficient information for an independent researcher to reproduce the reported results? If no, explain.

Yes.

Open Materials Badge

Please provide the URL, DOI, or other permanent path for accessing the materials in a public, open access repository.

Scrivener, C. L., Malik, A., Lindner, M., & Roesch, E. B. (2020, May 24). Sensing and seeing associated with overlapping occipitoparietal activation in simultaneous EEG-fMRI. https://doi.org/10.17605/OSF.IO/W6BH3

Is there sufficient information for an independent researcher to reproduce the reported methodology? If no, explain.

Yes.

Preregistered Badge

Please provide the URL, DOI, or other permanent path to the registration (and, if applicable, the analysis plan) in a public, open access repository.

Scrivener, C. L., Malik, A., Lindner, M., & Roesch, E. B. (2020, May 24). Sensing and seeing associated with overlapping occipitoparietal activation in simultaneous EEG-fMRI. https://doi.org/10.17605/OSF.IO/W6BH3. Specific path to the file: https://osf.io/tj84r/

Was the plan preregistered prior to the examination of the data or observing the outcomes? If no, explain.

Yes.

Were there additional registrations for the study other than the one reported? If yes, provide links and explain.

No.

For Preregistered and Analysis plan badge: were there any changes to the preregistered analysis plan for the primary confirmatory analysis? If yes, explain.

We ran an additional conjunction analysis after seeing the results from the preregistered fMRI analysis. This is clearly documented as an additional analysis step in the manuscript.

For Preregistered and Analysis plan badge: are all of the analyses described in the registered plan reported in the article? If no, explain.

No. We pre-registered a comparison between EEG data recorded inside the MRI scanner, which is described in the current paper, with data collected outside of the MRI (reported in a previous paper; Scrivener et al., 2019). However, we have decided to report this in a separate paper given the already long manuscript for this current submission. We also intend to run a more comprehensive comparison between the two data sets that we had originally pre-registered.
